# Heart failure patients monitoring using IoT-based remote monitoring system

**DOI:** 10.1038/s41598-023-46322-6

**Published:** 2023-11-06

**Authors:** Muhammad Umer, Turki Aljrees, Hanen Karamti, Abid Ishaq, Shtwai Alsubai, Marwan Omar, Ali Kashif Bashir, Imran Ashraf

**Affiliations:** 1 Department of Computer Science and Information Technology, The Islamia University of Bahawalpur, Bahawalpur, 63100 Pakistan; 2 Department College of Computer Science and Engineering, University of Hafr Al-Batin, 39524 Hafar Al-Batin, Saudi Arabia; 3 Department of Computer Sciences, College of Computer and Information Sciences, Princess Nourah bint Abdulrahman University, P.O.Box 84428, 11671 Riyadh, Saudi Arabia; 4 Department of Computer Science, College of Computer Engineering and Sciences, Prince Sattam bin Abdulaziz University, P.O. Box 151, 11942 Al-Kharj, Saudi Arabia; 5 Information Technology and Management, Illinois Institute of Technology, Chicago, USA; 6 Department of Computing and Mathematics, Manchester Metropolitan University, Manchester, UK; 7 Woxsen School of Business, Woxsen University, Hyderabad, 502 345 India; 8 Department of Computer Science and Mathematics, Lebanese American University, Beirut, Lebanon; 9 Information and Communication Engineering, Yeungnam University, Gyeongsan, 38541 Korea

**Keywords:** Pathology, Computer science

## Abstract

Intelligent health monitoring systems are becoming more important and popular as technology advances. Nowadays, online services are replacing physical infrastructure in several domains including medical services as well. The COVID-19 pandemic has also changed the way medical services are delivered. Intelligent appliances, smart homes, and smart medical systems are some of the emerging concepts. The Internet of Things (IoT) has changed the way communication occurs alongside data collection sources aided by smart sensors. It also has deployed artificial intelligence (AI) methods for better decision-making provided by efficient data collection, storage, retrieval, and data management. This research employs health monitoring systems for heart patients using IoT and AI-based solutions. Activities of heart patients are monitored and reported using the IoT system. For heart disease prediction, an ensemble model ET-CNN is presented which provides an accuracy score of 0.9524. The investigative data related to this system is very encouraging in real-time reporting and classifying heart patients with great accuracy.

## Introduction

Technology has brought about significant transformations across various domains, revolutionizing the world as we know it. It has enhanced communication, business operations, education, healthcare, and much more. Digital technology has played a pivotal role in breaking down geographical barriers, expanding our horizons, and facilitating business activities. Particularly in the wake of the COVID-19 pandemic, modern Intelligent applications have gained immense popularity and are being used for diverse purposes, including medical care. Additionally, the Internet stands as a remarkable technological achievement^1^, serving as the backbone that connects digital technologies and makes them accessible worldwide. Social media platforms have emerged as influential sources of information, particularly impacting the younger generation. Portable technologies provide instant access to a wealth of data through social media platforms IoT has rapidly emerged as a transformative field in computer science, significantly impacting various aspects of our world.

Concepts such as smart cars, smart-resource-based cities^2^, smartphones, smart driving, smart homes^3^, smart driving^4^, and smart farming^5^ have revolutionized the way people live and work worldwide. Smart devices play a crucial role in these smart homes and cities, enabling control over various aspects of daily life. Soon, smart devices are expected to track human activities in real-time and gather data using internal body sensors^6^. This data can be transmitted to medical experts, allowing for real-time monitoring of human health. IoMT which stands for Internet of Medical Things, specifically focuses on IoT-based health equipment integration^7^. AI combined with wearable tech has opened new horizons in the IoMT domain^8^, creating novel possibilities in the healthcare field to drive innovative developments. Given the significance of accurate and timely diagnosis and treatment in healthcare, further efforts are required to bridge existing gaps and create intelligent systems for health monitoring.

**Figure 1:**
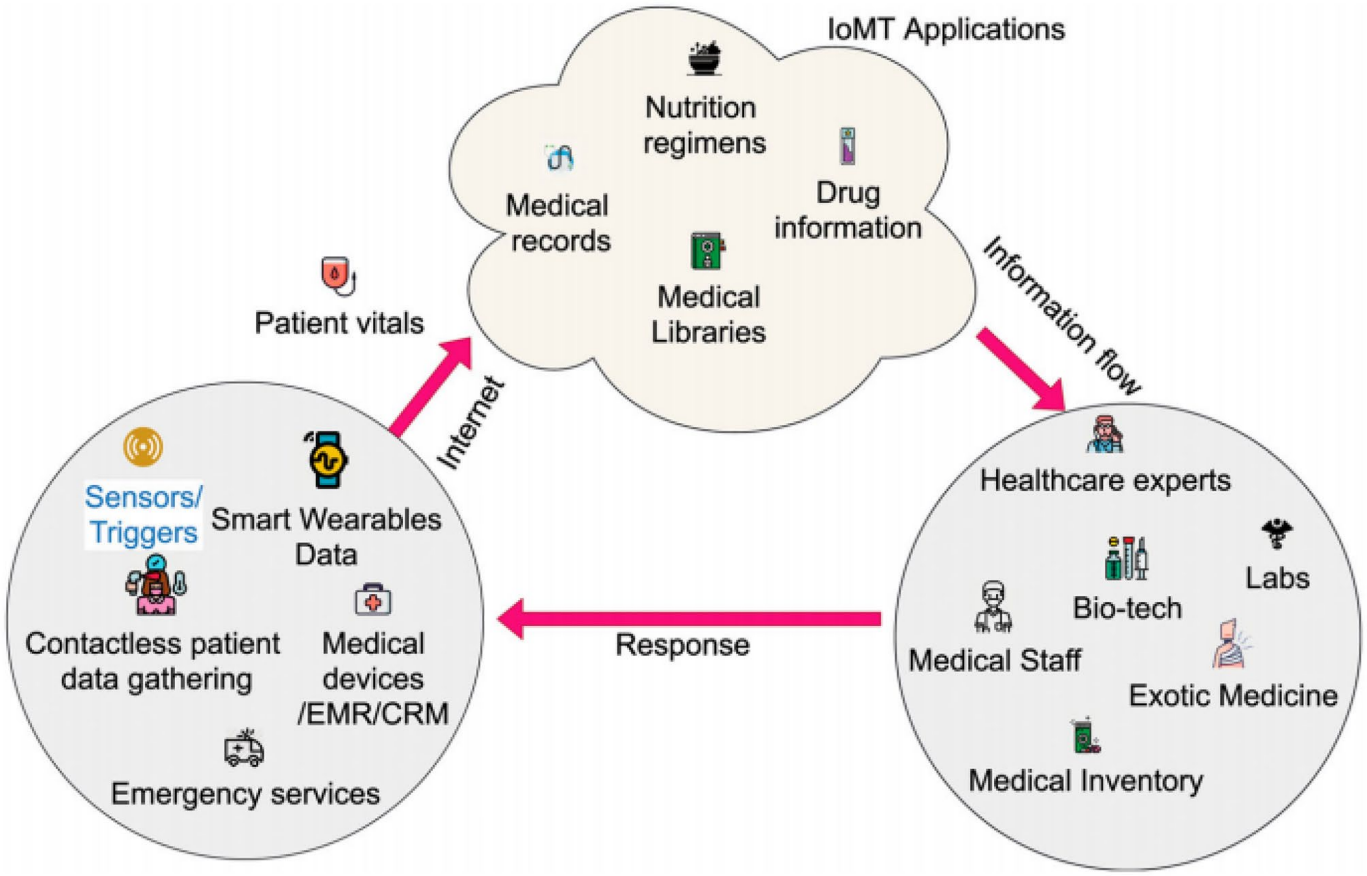
Mechanism of IoMT^10^.

Developed countries are experiencing a transformation in the medical field through the integration of Information Technology (IT). Conventional medical systems often suffer from overcrowding, high energy consumption, and overwhelming routine workloads, leading to service delays. In the realm of the Internet of Medical Things (IoMT), wearable sensor devices are interconnected with healthcare providers’ smart devices, enabling real-time tracking of patients’ health records and facilitating personalized treatment^9^. Patients’ health can effectively and affordably be monitored powered by IoMT. Limited staff and resources in third-world countries are under pressure and pose a significant challenge to the medical systems due to ever-increasing chronic diseases. Emergency response time is of vital importance when it comes to saving lives in the medical field. IoMT functioning is illustrated in Fig. 1 within the physical environment.

AI is the hottest computer domain that endows machines with intelligence, enabling smart devices to operate autonomously without human intervention. Smart devices leverage AI capabilities to establish seamless connections and exhibit creative functionality. Through AI processes, vast amounts of data collected from smart devices are analyzed to uncover concealed patterns^11^. Furthermore, AI processes generate recommendations to enhance system performance. AI and ML can aid in solving multiple complex problems in almost all walks of life. AI and ML are employed in computational systems to tackle dynamic challenges and improve various aspects of life. The integration of IoT, AI, and ML has the potential to revolutionize people’s daily lives and interactions. Within the medical field, sensors and smart devices accumulate extensive datasets. ML and AI algorithms are applied to identify fundamental trends and facilitate the diagnosis of different diseases.

AI-based solutions offer medical staff a convenient way to handle large datasets and provide proactive suggestions for disease prevention. The fusion of artificial intelligence methods with health diagnosis techniques AIoMT, (Artificial Intelligence of Medical Things), has proven beneficial in the healthcare sector. The primary objective of AIoMT is minimizing healthcare expenses and hospital admissions^12^. As the population continues to grow, traditional methods of medical diagnosis are insufficient to meet the increasing demands. Given the scarce resources and the challenges posed by population growth, finding effective resource management solutions becomes a top priority. Proper diagnosis and the subsequent improvement of treatment methods contribute to effectively managing infectious disease outbreaks. IoMT has significantly improved the site of infections in^13^. The authors examined cybersecurity threats associated with IoMT architecture, as well as the security criteria essential for IoMT^14^. Several initiatives are discussed in^15^ to enhance security requirements, address design challenges, and explore security techniques aimed at bolstering the security of IoMT.

Post-COVID-19, there is a pressing need for a real-time diagnosis remote healthcare system to address various diseases. To enhance healthcare facilities, collaborative efforts between academia and industry are essential to tackle these challenges. Although recent initiatives have focused on designing smart point-of-care (PoC) devices and smart bed concepts, additional endeavors are required to accommodate the growing population. As per the World Health Organization (WHO), an alarming 17.9 million deaths are reported annually due to heart problems^16^. Cardiac disease is a serious healthcare challenge and typically arises from arterial narrowing. In underdeveloped countries, heart failure is a major fatality contributor. While elderly individuals are commonly affected, heart disease can also afflict younger patients. In the United States, heart disease accounts for one out of every nine deaths^17^. The primary symptoms of heart disease include chest pain and fatigue, although it can also manifest without noticeable symptoms. It is crucial to improve a distant health monitoring system specifically for individuals with heart conditions, ensuring continuous monitoring of their activity. In emergency situations, these remote health monitoring systems can offer immediate assistance. IoMT is of great significance in medicine for remote areas where medical emergency response time is not up to the mark. Early discovery and treatment of diseases are helpful in saving precious lives. Moreover, remote health monitoring minimizes the costs associated with routine diagnoses by eliminating travel expenses and other medical costs.

The IoMT (Internet of Medical Things) represents a complex intersection of IoT and medical technology, presenting numerous challenges. Given the critical nature of healthcare and the preservation of human life, a modern IoMT-based AI-integrated modern distant healthcare monitoring system is a need of the hour. In order to comply with medical standards, software systems in medical contexts require meticulous and comprehensive verification. The IoMT-based AI system must comply with the following:

- *Security* Security of all internet-based ventures is a must and a healthcare monitoring system for cardiac diseases, based on AI technologies is no exception^18,19^. So Security of data must be ensured by having a reliable remote healthcare monitoring system. It should possess a robust defense against potential cyber-attacks from other devices. Precise readings of such systems are of vital importance and there should be no compromise on the cyber security of such systems as any counterfeit reading can cost precious human lives.
- *Reliablity* All digital technology is software driven and medical software systems are no exception in this context. So the reliability of such software systems is of utmost importance^20,21^. A system lacking reliability cannot be employed in any domain, especially when it involves the medical history, confidentiality, privacy, and related credentials of patients. It is essential to address internal implementation issues and external factors to guarantee accurate measurements. To prevent any potential harm, every system should incorporate an auto-correction algorithm to promptly rectify any errors or discrepancies.
- *Safety* The remote monitoring software should prioritize cost-effectiveness while minimizing its environmental impact. The system must be designed in a human-friendly manner^22,23^, ensuring that it does not have any adverse effects on humans. Designers, users, and medical operators should be trained enough to use the system without facing any negative consequences.

This study presents the design of a remote monitoring software system that leverages IoMT and utilizes AI techniques. The system collects spontaneous human data and applies AI algorithms in collaboration with biomedical experts to classify cardiac diseases and patients based on historical data and hidden patterns. By analyzing the live instantaneous patient data, the system detects the presence of heart disease. This remote monitoring software system offers valuable support for early diagnosis and eliminates the need to reach a hospital. The proposed system is of literature documented system’s standard in relation to its performance as demonstrated by various trials. The primary contributions of this study are given here

- A comprehensive overview of the current body of literature on the IoMT is provided, particularly highlighting the primary applications of IoMT in healthcare. Furthermore, key challenges and difficulties in implementing IoMT are discussed.
- An intelligent health monitoring system is proposed using the IoT paradigm that collects and analyzes data from individuals with heart conditions. In addition, machine and deep ensemble learning ensemble model ET-CNN is incorporated in the IoMT system to efficiently detect and identify patients that need emergency help for heart care.
- The proposed system’s performance is analyzed through multiple experiments, and a thorough analysis of its effectiveness is conducted by comparing it to current state-of-the-art methods.

The remainder of the paper is structured as follows: In section "Related work", an overview of the previous literature is presented. Section "Introduction and background of IoMT" elucidates the functioning of IoMT. Section "Proposed framework of intelligent healthcare system" outlines the dataset utilized here and presents the proposed intelligent framework, along with an explanation of the different algorithms employed. Experimental details and evaluation parameters are provided in section "Experimental results". Finally, section "Conclusions" concludes the paper.

## Related work

In the modern era, numerous technologies have emerged to facilitate the monitoring of medical data. The rapid progress in this field has been driven by the development of sensors, which enable instantaneous data collection and monitoring. Smart devices are equipped with various sensors and have become valuable tools for data collection. For instance, a ring-based sensor technique is utilized to screen patients^24,25^, where a wearable ring with a sensor enables real-time cardiac monitoring. Similarly, in^26^, an ear-based sensor technique is employed for uninterrupted monitoring of individuals with heart conditions. Being compact in size and easily wearable by different age groups, these sensors facilitate the monitoring process.

Rapid response is crucial for heart patients during emergencies, as delays in early diagnosis and ineffective methods can lead to fatal outcomes. In^27^, the authors propose a technique that leverages IoT technology and cloud resources for early diagnosis. Managing and storing medical data pose significant challenges in the healthcare field, but by integrating IoT technology with cloud infrastructure, which offers extensive storage capacity, substantial improvements can be achieved. An energy-efficient protocol for healthcare applications and gadgets has been developed in a related study^28^ employing coding with dynamic channels combining multiple-layer access with physical access. The primary objective is energy optimization and enhancing nodes’ network lifetime.

The COVID-19 pandemic posed a significant risk to the health of frontline healthcare workers, necessitating extra precautions in patient care. Remote health monitoring applications played a crucial role in enabling medical staff to effectively diagnose patients. These applications’ implementation is done using IoT-based devices^29,30^. Real-time public surveillance is done at various public locations using image-processing applications. Further analysis can be done through IoMT-based data retrieval by medical professionals^31^.

IoMT technology has witnessed the presentation of various approaches and systems utilizing IoT connectors, as demonstrated in Table 1. The health-monitoring system was proposed by Jain et al.^32^ that leverages near-infrared spectroscopy and machine learning (ML) techniques for glucose level analysis. The use of IoMT edge devices for human health monitoring has become increasingly prevalent, enabling swift and accurate outcomes. The proposed glucose level detector exhibits a significantly reduced error ratio related to current approaches in the literature. A methodology for classifying diseases is introduced by Shui-Hua Wang et al.^33^ including tuberculosis, COVID-19, pulmonary diseases, and pneumonia. This method enhances the accuracy of disease diagnosis by medical professionals and exhibits superior performance in detecting various illnesses. In^34^, remote-controlled ambulance service is designed in an approach by the authors. Real-time results are obtained using artificial intelligence (AI) approaches, which prove beneficial in medical applications that demand prompt and accurate responses. Similarly, Harshal Arbat et al.^35^ developed a methodology that utilizes a smart health band to monitor heart patients. The band measures heart rate data, which is then analyzed to assess the patient’s health status.

**Table 1:** Applications of IoT and their connectors.

References	Standards	Purpose
^36^	Bluetooth	They used for the detection and monitoring of the sounds of the heart
^37^	Bluetooth	They used it for the monitoring of fitness during the physical activity
^38^	Wifi	They used it for the health condition monitoring remotely
^39^	3G	They used it for faster data communication during emergency situations
^40^	Satellites	They used it for the patents tracking in case of emergency situations
^41^	NFC	They used it for the monitoring of the Home for the persons who are not enough skillful
^42^	2G, 3G	They used it for the real-time skincare
^43^	RFID	They used it for the quick and timely tracking and localization of the medical instruments

In today’s world, smart devices are vigorously used in the healthcare domain to aid professionals in analyzing human health data. In^44^, a mobile application-based approach is devised for monitoring human health. When mobile apps are integrated with cloud computing and IoT, the proficiencies of health monitoring systems can be greatly enhanced. Ample storage space to host large volumes of IoT-based data is the hallmark of cloud technology to provide processing services for rendering. This method is also able to assess the stress level of the body by measuring the brain signals. Michael Fischer et al.^45^ presented a method that even enables non-professionals to get information regarding certain diseases. Bot-based diagnosis of the patients is done by providing instructions to the bot. Integrating smart devices with bots enhances the provision of healthcare services. Although the technique’s accuracy may be lower compared to other methods, it represents a significant stride toward automated disease diagnosis. A comprehensive overview of IoT-based studies can be found in Table 2.

**Table 2:** Previous IoT base state-of-the-art systems.

References	Year	IoMT	Limitations
^44^	2016	A system for electrocardiogram (ECG) that operates on the cloud and enables remote access	Limited in assessing stress level through brain signals
^46^	2016	A robust healthcare system that prioritizes patient security	Lack of integration with smart devices
^47^	2016	An advanced healthcare system utilizing intelligent technology to benefit patients in the field of medicine	Limited automation in disease diagnosis
^48^	2016	A system that uses the Internet of Things (IoT) to detect and recognize kidney anomalies	Limited scope beyond kidney anomaly detection
^49^	2016	Healthcare empowered by the Internet of Things (IoT) technology	Lack of specific application focus
^50^	2016	A mobile healthcare system that leverages the capabilities of the Internet of Things (IoT) for enhanced medical care	Limited accuracy in disease diagnosis
^51^	2022	National Instrument myRIO is used for smart data.	Scalability for handling a larger number of patients is not addressed
^52^	2022	ICU monitoring systems designed using IoT devices.	The need for user training, and scalability concerns
^53^	2023	Remote monitoring of patients using heath sensors	Data protection standards is essential but may pose challenges

In a similar fashion, a combination of IoMT devices, cloud technology, and IoT sensors is used in developing a comprehensive healthcare system to cater to the needs of heart patients^54^. The system monitors body temperature, oxygen levels, and eye movement as indicators of heart disease. Similarly, in^55^, the authors devised a model for heart disease monitoring. Early diagnosis of cardiac patients is enabled in an ensemble model and utilizing sensor data within a fog computing environment. Cardiac disease is identified by using an adaptive Neuro-Fuzzy Inference System having multiple kernel learning employed to identify heart disease^56,57^. Despite better results, it has a computational complexity of a higher degree. A programmed method is used to segregate individuals having low heart failure risk from higher risk heart failure individuals^58^. The authors utilized the CART (Classification and Regression Tree) technique and achieved sensitivity and specificity values of 93% and 63%, respectively. Authors applied Arduino UNO with various IoT sensors to monitor the heart^59^. An inclusive overview of advanced cardiac disease studies can be found in Table 3.

**Table 3:** Previous studies related to heart disease.

Reference	Approach	Dataset	Challenges
^56^	Multi-Kernel Learning and Neuro-Fuzzy Inference	KEGG Metabolic Dataset	inappropriate performance evaluation metrics
^60^	BiGRU, BiLSTM, CNN ensemble	Cardiac Disease Dataset	The study uses the dataset is not a standard dataset
^61^	RF, XGB, and GBC	Multiple heart disease datasets such as Cleveland, Hungarian, Z-Alizadeh Sani Dataset	Stacking three models introduces higher complexity and cost to the model
^62^	Fisher technique and Generalized Discriminant Analysis	two datasets NSR-CAD and SR-CAD	The training process on large datasets for heart rate variability needs improvement due to the existing lack of training
^63^	NB, k-NN, RF, and DT	Multiple heart disease datasets such as Cleveland, Hungary, VA Long Beach, Switzerland Datasets	It is important to conduct further experimentation with different combinations of models and feature choices to explore potential enhancements
^64^	LSTM and RNN	IoT cloud data from UCI machine learning repository’s Cleveland and Hungarian datasets	The challenges include managing vast amounts of patient data, integrating Internet of Things (IoT) devices, utilizing deep learning for accurate disease diagnosis, ensuring data quality, addressing privacy and security concerns, achieving early disease recognition, and extracting meaningful features from the data
^65^	Bi-LSTM	data collected from IoT sensors, electronic clinical data (ECD)	Key challenges include managing and processing vast healthcare data, ensuring data quality, maintaining privacy and security, early disease detection, and personalized healthcare

## Introduction and background of IoMT

The Internet of Medical Things (IoMT) is a network of interconnected medical devices and applications that collect, transmit, and analyze healthcare data^66^. IoMT leverages the Internet of Things (IoT) technology to improve healthcare delivery, patient monitoring, and medical research. Here’s how IoMT works:

1. **Medical Devices and Sensors:** IoMT relies on various medical devices and sensors equipped with sensors and communication capabilities. These devices can be wearables (like smartwatches and fitness trackers), implantable devices (like pacemakers), or standalone monitoring equipment (like blood pressure monitors or glucometers).
2. **Data Collection:** Medical devices continuously collect health-related data from patients or individuals. This data can include vital signs (e.g., heart rate, blood pressure, temperature), activity levels, glucose levels, ECG readings, and more. The sensors convert these physical measurements into digital data.
3. **Data Transmission and Storage:** The collected data is transmitted securely over the internet or a dedicated network to a central location. This central location can be a cloud server, a hospital’s data center, or a healthcare provider’s network. Data storage often involves rigorous security measures to protect sensitive healthcare information.
4. **Remote Monitoring:** IoMT enables remote patient monitoring, allowing healthcare providers to keep tabs on patients’ health status without the need for in-person visits. This is particularly valuable for chronic disease management and post-surgery recovery.
5. **Patient Empowerment:** IoMT empowers patients by giving them access to their own health data. Patients can monitor their progress, set health goals, and make informed decisions about their well-being.
6. **Data Analysis:** Healthcare professionals and algorithms analyze the collected data. Machine learning and artificial intelligence (AI) are often used to identify trends, anomalies, or patterns in the data. This analysis can help in diagnosing conditions, monitoring patients, and making treatment decisions.

IoMT holds immense potential to revolutionize healthcare by improving patient outcomes, reducing healthcare costs, and enhancing the overall quality of care^67^. However, it also poses challenges related to data security, interoperability, and regulatory compliance that must be carefully addressed to ensure its successful implementation.

Fundamentally, Internet of Medical Things (IoMT) systems operate by integrating numerous technologies. The selection of these technologies depends on the nature of the desired task, latency requirement, security needs, etc.

### Communication technologies

IoMT can use several communication technologies including Ethernet, Bluetooth, Zigbee, Wi-Fi, near-field communication, and satellite communication. Ethernet can use a local area network (LAN) or a wide area network (WAN). The Ethernet connection is known for its reliability, high-speed capabilities, and enhanced security measures. Typically, Ethernet operates at a standard speed of 10 Mbps. Bluetooth, on the other hand, is more suitable to enable wireless communication among electrical appliances over short distances. It operates through short-range radio frequencies. Bluetooth devices typically function within a range of approximately 10 m, making them suitable for medical equipment, where close proximity communication is often required.

Intermediary devices based on mesh networks are used by Zigbee devices for the transmission of data over extended distances, enabling communication with devices located farther away. Compared to Bluetooth, Zigbee technology exhibits faster data transmission capabilities. Today, Zigbee technology is predominantly used with IoT technology. It also provides extra security with 128-bit encryption for secure data transmission.

Wi-Fi is more suitable to establish a link between computers, tablets, cell phones, and others. Wi-Fi adheres to the IEEE 802.11 standards and operates over a wide communication range. To increase the number of connected devices to a certain Wi-Fi network from an extended range, an access point or Wi-Fi range extender is coupled to the router allowing increased connectivity and coverage. Near-field communication (NFC) is employed for wireless connectivity over short distances, typically within a range of 4 cm. NFC enables swift and effortless transmission of data with a simple touch between gadgets. NFC technology operates either in active mode without pairing, or in passive mode requires pairing for communication between devices. Contrary to NFC which provides the lowest range, satellite communication provides the longest range. Satellites are positioned at high altitudes around Earth. In modern times, satellites are extensively employed in weather forecasting, telecommunication, and geopositioning. They play a crucial role in analyzing regions that are inaccessible by conventional means. In the recent past, satellites have also been instrumental in capturing and transmitting solar system images.

### Challenges of IoMT

Due to its sensitive nature, the medical domain requires a higher level of privacy and confidentiality of the patient data^10,68^. In the context of IoMT, wireless technology is predominantly utilized, raising significant concerns about data privacy. Several key challenges pertaining to this issue are discussed below.

1. Robust cybersecurity in IoMT is a big challenge. Data privacy must be ensured for the sensitive data of patients^69,70^. To tackle privacy and security, blockchain technology is one feasible solution^71^.
2. Interoperability poses a challenge in utilizing IoMT systems. The complexity of exchanging data makes interoperability difficult between multiple IoMT-based systems^72,73^. Standardized interfaces are needed to resolve this issue.
3. The integration of sensors and medical equipment can be costly to upgrade and maintain. The inclusion of affordable and maintenance-free sensors can stimulate the development of IoMT-based devices and enhance their widespread adoption^74^.
4. The power consumption of IoMT devices is also a big concern. Most IoMT sensors, require backup solutions or high-capacity batteries^75^. Sustainable medical appliances are the need of the hour.
5. Biomedical sensors involve several semiconductors that contain earth elements and hazardous chemicals, potentially causing adverse environmental effects^76^. Centralized overseeing and supervising sensor production needs to be ensured.

### Vulnerabilities of IoMT

The IoMT has shed light on critical security challenges in the healthcare sector. IoMT, while promising significant advancements in patient care and monitoring, presents vulnerabilities that can have far-reaching consequences^77^. One of the foremost concerns is the potential for data breaches and unauthorized access to sensitive medical information. Weak authentication mechanisms and inadequate encryption can expose patient records to malicious actors. Additionally, device vulnerabilities, often stemming from outdated software or firmware, create opportunities for cyberattacks that could compromise patient safety and privacy. Furthermore, the reliance on interconnected networks makes IoMT susceptible to network-based attacks, such as man-in-the-middle attacks or denial of service incidents, threatening the availability and integrity of healthcare services. As healthcare organizations continue to embrace IoMT technologies, understanding and addressing these vulnerabilities are imperative to ensure the security and trustworthiness of healthcare systems and the protection of patient data.

### Advantages of IoT-based healthcare monitoring systems

The development of healthcare monitoring systems has garnered significant attention from researchers and medical experts. Various effective research initiatives have been undertaken in the medical field, with many more currently in progress^78^. The growing numbers of elderly and chronic conditions patients have led to an increasing gap between those receiving healthcare and those in need of treatment. Traditional healthcare, primarily provided in hospitals, often falls short in meeting the needs of the elderly and individuals with impairments^79,80^. However, the Internet of Things (IoT) presents a practical and effective solution for real-time health monitoring of the elderly through sensor data and telecommunications. The combination of IoT and smart technology has the potential to enhance and expand healthcare services. Scientists have created a range of emergency systems that make use of sensors and advanced wireless communication, specifically designed to monitor the health of elderly individuals. By having vital signs and other related health data, valuable insights into general health and potential risks can be obtained^81^.

### Significance of IoT utilization in healthcare

IoT has revolutionized the healthcare industry by transforming how healthcare is delivered. We are now entering a new era where applications, technologies, and healthcare providers are interacting in unprecedented ways. The IoT enables the creation of integrated healthcare networks, offering valuable insights and tools. Previously time-consuming and error-prone healthcare processes can be automated with the aid of AI and IoT, once dependent upon human involvement. For example, many hospitals have implemented networked equipment to regulate ventilation and temperature in operating rooms. The potential benefits of IoT in healthcare are numerous, but a few key ones are enlisted here:

- Reduced chance of human errors.
- Overcoming limitations of physical visits and distance.
- Streamlined record-keeping.
- Early detection and treatment of chronic disorders.
- Enhanced management of medicine.
- Prompt medical focus.
- Better treatment outcomes.

The integration of IoT technologies in healthcare holds great promise for improving efficiency, accuracy, and patient care across various aspects of the healthcare ecosystem.

## Proposed framework of intelligent healthcare system

**Figure 2:**
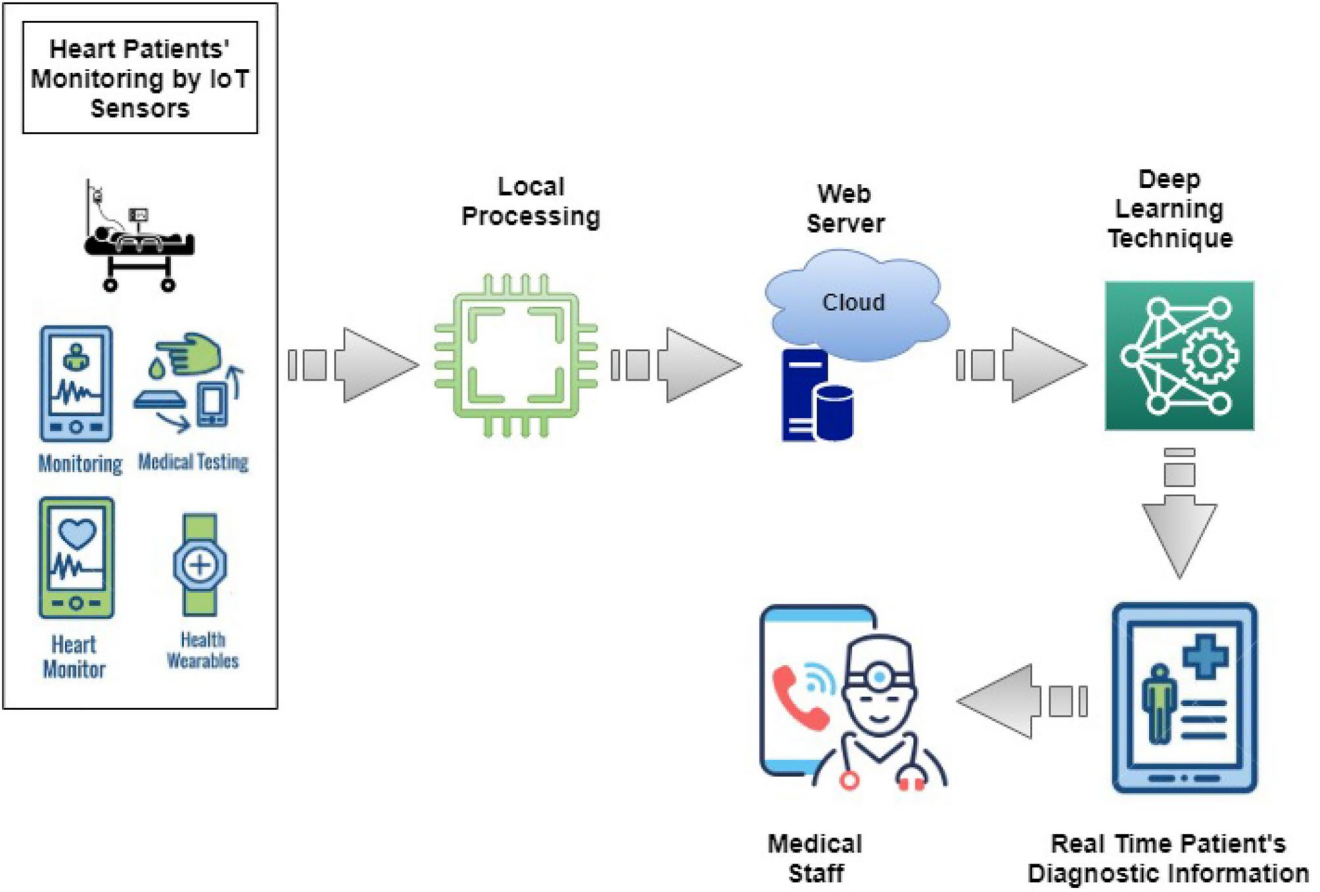
Proposed IoT-based monitoring system workflow.

This section introduces a healthcare platform based on IoT which is specifically designed for individuals with cardiac failure. The framework incorporates artificial intelligence (AI), cloud services, and IoT technologies. Figure 2 illustrates the proposed system layout for intelligent healthcare^82^. By utilizing IoT devices, this framework enables medical personnel to conveniently monitor their health. Leveraging cloud-based and IoT technologies, medical records of patients suffering from heart failures can be retrieved anytime from around the globe. The Algorithm 1 demonstrates the comprehensive flow of the proposed architecture.

**Algorithm 1:**
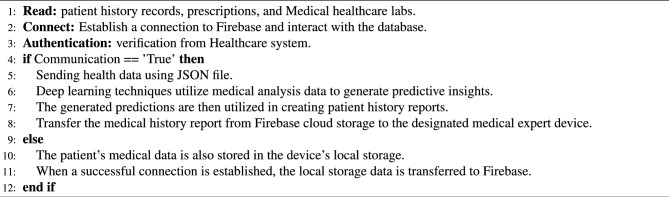
Steps carried out for the IoT-based heart monitoring system.

Zigbee and Firebase authentication is used to implement security measures in an intelligent healthcare system based on an IoT. During data transmission, 128-bit encryption is applied as a token to the JSON file. Firebase authentication is used to verify the device token by generating a token containing customs token claims and exact credentials. Firebase custom token and device-generated 128-bit token verify the user identity during real-time data exchange between devices. The authorization process follows authentication using Firebase’s general Security rules. The security mechanism of Firebase can be summarized as a three-step security measure, outlined as follows:

- The device token is the guarantee that a particular request has originated from a particular device but it does not carry any valuable information.
- A custom token includes all of the user’s data except for their profile information. Additionally, Firebase servers cannot inherently rely on this token since the service account used by our Cloud Function may not always have authorization. For example, there may be situations where we decide to invalidate the token or change its encryption key.
- The assertions within a custom token are validated using the signInWithCustomToken API. Afterward, the backend generates a Firebase ID token that includes the user profile and acts as undeniable proof that the bearer has authorization to carry out actions on behalf of the user. This token, which remains valid for 1 hour, cannot be invalidated.

Figure 2 depicts the mechanism by which intelligent systems facilitate the transmission and updating of patient data within the Internet of Things (IoT). The equipment to be used is selected on a patient care facility and hospital-specified basis. The proposed construction allows for the real-time monitoring of patient data, enabling prompt access to emergency medical care for patients. Since the data is stored in the cloud, healthcare professionals can remotely access the information and provide guidelines by taking into account the patient’s condition.

The core aim of this intelligent system is to improve patients’ survival chances by offering an affordable, reliable, dependable, and simple monitoring system for heart-related problems. The suggested system collects data from various sources and transmits it to the cloud for processing using deep learning and ML models. This helps healthcare professionals to access structured data, facilitating comprehensive investigation and analysis.

### Dataset

The dataset used in this section has been from the UCI-ML repository comprising heart failure records related to the medical domain^83^. This specifically pertains to patients with serious heart failure, encompassing 11 clinical features that were collected during follow-up. Among 299 dataset records, 194 were male patients and 105 were female patients. The features of the dataset are outlined in Table 4.

**Table 4:** Heart disease dataset details.

Serial no.	Attribute	Description	Range	Measured In
1	Time	It represents the development age	4–285	Days
2	Events	It represents the death of the patent during the development period	0,1	Boolean
3	Gender	It represents the sex of the patient	0,1	Binary
4	Smoking	It represent that whether the patient smokes or not	0,1	Boolean
5	Diabetics	It represents that whether the patient is diabetic or not	0,1	Boolean
6	BP	It represents whether the patient has blood pressure or not	0,1	Boolean
7	Anaemia	It represents whether the patient has RBC (Red blood cell) deficiency or not	0,1	Boolean
8	Age	It represents the age of the patient.	40–49	Years
9	Sodium	It represent the level of sodium in the patient body	114–148	mEq/L
10	Creatinine	It represent the level of Creatinine in patient body	0.50–9.40	mg/dL
11	Platelets	it represent the Blood platelets level in patient body	25.01–850	Kiloplatelets/mL

### Deep learning model

Deep learning has emerged as a rapidly growing research area within the AI domain. The deep learning methods applied in data demonstration have yielded promising outcomes. Medical professionals found automated processes highly efficient and effective in diagnosis. DL is known for its ability to handle large volumes of data, eliminate manual feature extraction, and has gained popularity in medical data analysis.

#### Multilayer perceptron

When dealing with small training datasets, the Multi-Layer Perceptron (MLP) is often the preferred option due to its ability to produce rapid results and its reliability^16,84^. The MLP is composed of three layers: the input layer, the output layer, and the hidden layer. The hidden layer serves as a mediator between the input and output layers, facilitating the processing of neurons. The internal operations of the MLP involve multiplying the input neurons by their corresponding weights $w_{ij}$, and the output $y_j$ is determined by summing these values. This computation can be expressed using the following formula:

$$\begin{aligned} y_{j}=f\left( {\sum {w_{ij}*O_{i}} }\right) , \end{aligned}$$

In this equation, the weights *w* are assigned to the gradient descent algorithm, while *O* represents the hidden layers.

### RNN

RNN is considered to be an optimal choice when sequential neural networks are discussed^16^. In RNN processing, a weighted sequence from one neuron’s input is fed to other neurons resembling the sequential arrangement of words in a sentence. RNNs are drafted to generate sequences and predict the next word in the loop, thus capturing the temporal dependencies within the data.

### Convolutional neural network

**Figure 3:**
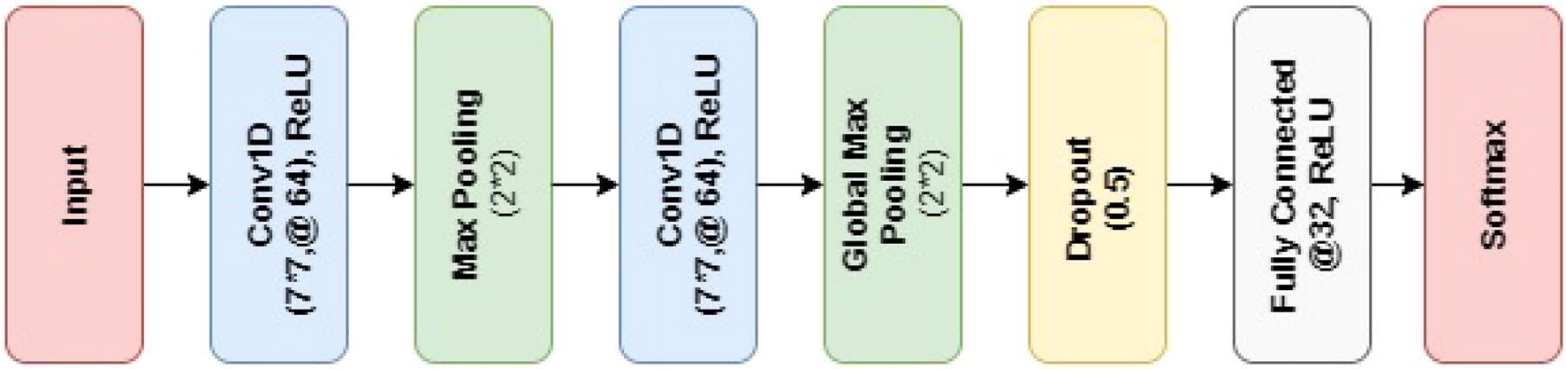
Layers structure of the proposed CNN model.

CNN (Convolutional Neural Network) is a powerful neural network model that excels at capturing intricate relationships among various data attributes. It is particularly well-suited for image analysis tasks, as it can effectively analyze input images, identify and rank different objects and features within the images, and distinguish them. A CNN comprises a node layer and a hidden layer including output and input layers. To achieve improved outcomes, this study employs a tailored CNN structural design^82^, as depicted in Fig. 3.

The proposed 8-layer architecture consists of 2 max-pooling layers, 2 dense layers, and 2 convolutions layers. In medicine, CNN has proven to be a game-changer approach for classification tasks. In the CNN model, the error function is denoted by Sigmoid, and the backpropagation algorithm is employed. CNN can easily classify various diseases including lung disease, brain tumors, and cardiac diseases. It is preferably used in the medical field for handling larger data. The pooling layer in CNN can either be average or maximum pooling. Maximum pooling is commonly used for extracting sharp features, while average pooling is employed for extracting flat features.

### Long short term memory

An enhanced version of RNN known as LSTM (Long Short-Term Memory) is particularly effective for processing long-term sequences. LSTM addresses the vanishing gradient problem often encountered by RNNs and surpasses them in performance. It has the capability to memorize specific patterns in data. An LSTM is composed of three gates: the input gate, the output gate, and the forget gate. These gates play crucial roles in the LSTM’s ability to control information flow. The representation of the word sequence is illustrated in Equations (1)–(3).

1 $$\begin{aligned} i_t= & {} \sigma (x_tU^i + h_{t-1} W^i + b_i) \end{aligned}$$

2 $$\begin{aligned} o_t= & {} \sigma (x_tU^o + h_{t-1}W^o + b_o) \end{aligned}$$

3 $$\begin{aligned} f_t= & {} \sigma (x_tU^f + h_{t-1}W^f + b_f ), \end{aligned}$$

In the given context, the variables can be defined as follows: $x_t$ is the input sequence, $h_{t-1}$ is the previous hidden state, *t*, $i_t$ is the input gate, which controls the flow of information, $o_t$ is the output gate, $f_t$ is the forget gate.

### Experimental design

**Figure 4:**
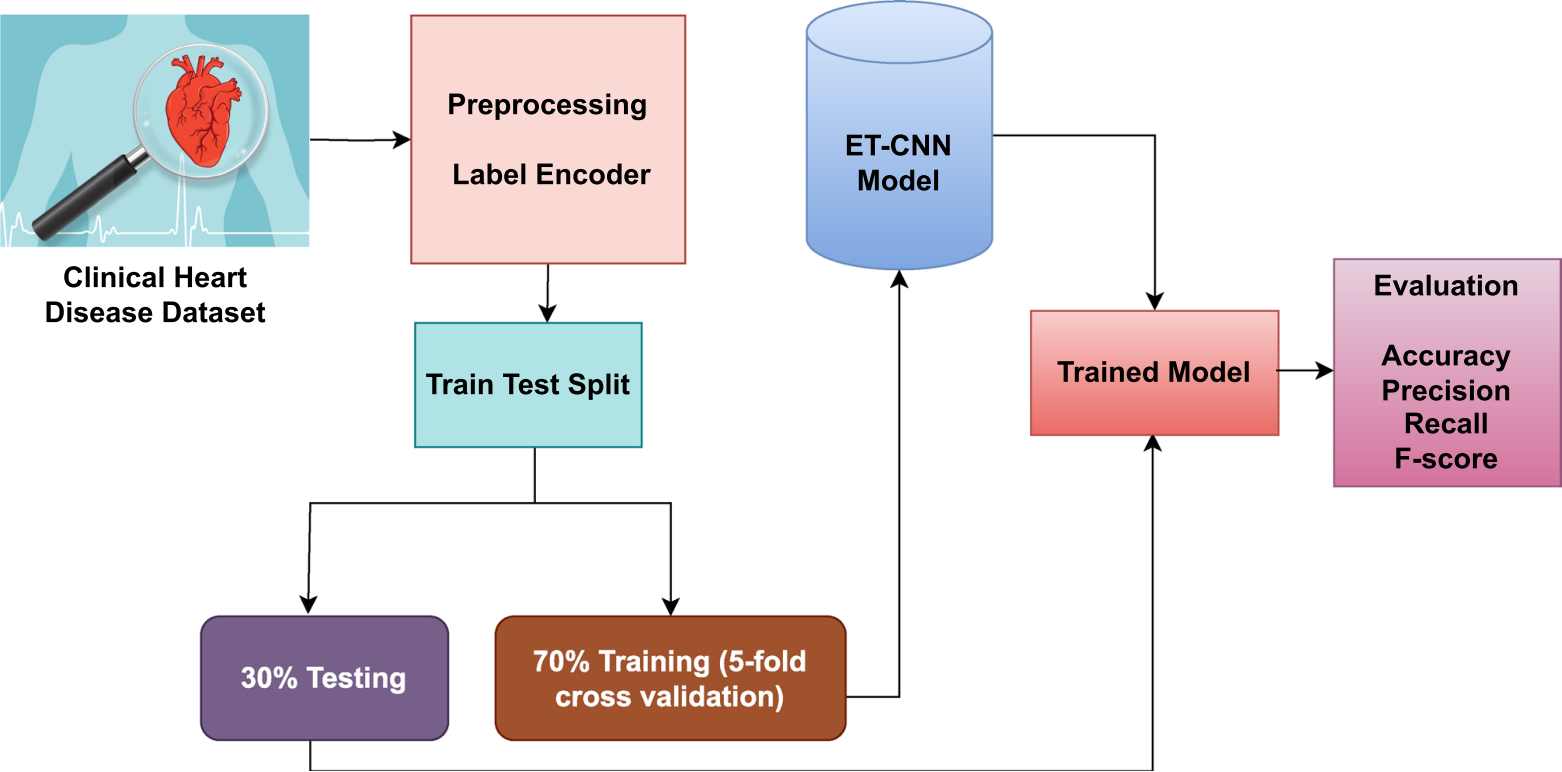
Workflow diagram of the proposed model.

In this study, a highly efficient health monitoring model is introduced specifically for monitoring patients with heart conditions. The model proposed in this research incorporates a machine-deep ET-CNN architecture, as visualized in Fig. 4. Furthermore, RNN, LSTM, and multilayer perceptron (MLP) models are also utilized. The MLP model comprises hidden input and output layers. The hidden layer functions as the processing layer, establishing connections between the input and output layers.

In the equation, *O* represents the hidden layer, and w denotes the weight value.

In the experiments, the performance of multiple-layer structures is evaluated, and it is found that hidden-layer structures yield favorable results. The hidden layer structure provides features from the cardiac failure dataset as input. The following parameter values were set: a learning rate of 0.01, a batch size of 256, a dropout rate of 0.2, and 25 epochs. Additionally, the CNN approach-based performance evaluation method is implemented to ascertain its effectiveness. The CNN-based approach employed a total of 8 layers. ET-CNN-based approaches having initial parameter values are listed in Table 5.

During the initial stage, the input dataset was the heart failure dataset. Specifically, 13 features from the heart failure dataset are selected to train the data, aiming at improving the accuracy of predictions. A timely and accurate diagnosis, alongside appropriate treatment, is crucial for patients with acute heart failure to enhance their chances of survival.

**Table 5:** ET-CNN models parametric values.

Parameter	Value
Number of tree	200
Node split size	10
No. of jobs	$-1$
Random state	52
Batch size	256
Epochs	25
Function	Binary cross entropy
No. of filters	5 $\times$ 64
Optimizer	Adam
Pooling	2 $\times$ 2

**Figure 5:**
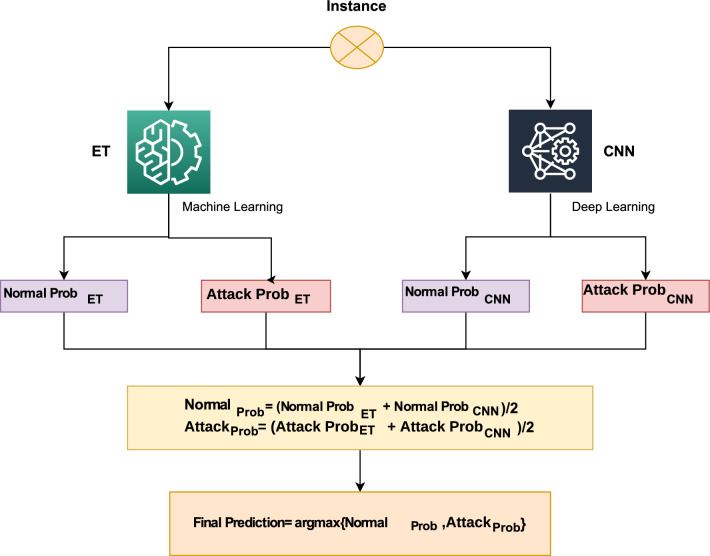
ET-CNN architecture.

Voting classifiers combine the results of various classifiers to make a final decision based on voting. There are two types of voting classifiers: Soft and hard voting. The percent weight of each classifier is computed by soft voting while classifiers’ results prediction is done using hard voting. For every entry, class probability multiplied by classifier weight and then averaged to determine the final result is predicted by this prototype. In our research, a voting classifier, an extra tree classifier (ET), and a convolutional neural network (CNN) are used in combination, outperforming other techniques for heart patient disease prediction. Algorithm 2 illustrates the methodology of the projected voting classifier, presented as follows:

4 $$\begin{aligned} \widehat{p}=argmax\left\{ {\sum _i^n}ExtraTreeClassifier_i, {\sum _i^n}Convolutional Neural Network_i \right\} . \end{aligned}$$

where ${\sum _i^n}ExtraTreeClassifier_i$ and ${\sum _i^n}Convolutional Neural Network_i$ predict the probability-based results for each test model by Extra Tree Classifier and Convolutional Neural Network, respectively. Extra Tree Classifier and Convolutional Neural Network instance’s probabilities are passed through soft voting criteria in Algorithm 2. Figure 5 shows the visual representation of the proposed ensemble model.

**Algorithm 2:**
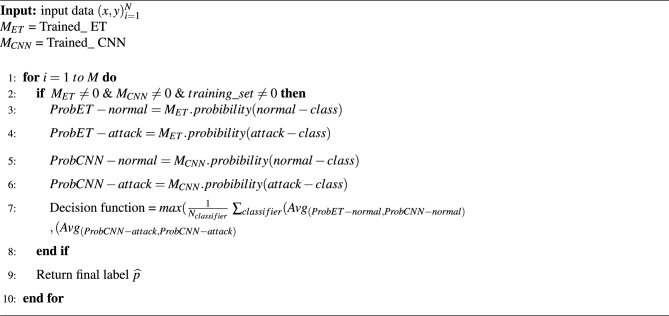
Ensembling of Extra Tree Classifier and Convolutional Neural Network Classifier (ET-CNN).

### Performance evaluation metrics

To assess the effectiveness of the proposed model, various performance measures are computed, such as precision, accuracy, and F1 score, among others. A model’s accuracy is of prime importance in the healthcare field because here accurate predictions actually matter. These performance measures help determine the reliability and effectiveness of the model in making accurate predictions, contributing to informed decision-making and improved patient outcomes.

## Experimental results

To evaluate the performance of the ET-CNN-based model, a dataset containing information from heart patients is used. Along with the deep learning algorithms MLP, CNN, LSTM, and RNN, machine learning algorithms were also employed for comparison purposes. A training-testing ratio of 70-30 was applied to all prototypes in order to assess their performance. The DL algorithms were implemented using Python libraries such as Keras and TensorFlow.

Table 6 presents the performance evaluation of DL models in terms of accuracy, recall, precision, and F1 scores. The ET-CNN model takes the lead, achieving a 0.9524 accuracy score, along with precision, recall, and F1 scores of 0.97 each.

The CNN model outperforms the other deep learning models in terms of performance. A comparative analysis was conducted with two other models mentioned in the study^85^. The referenced study conducted experiments under various situations, including oversampling through the SMOTE (synthetic minority oversampling technique). The SMOTE method is applied to both the ETC and the CNN models. The outcomes presented in Table 6 demonstrate that the CNN model performed better than ETC and CNN models employed with SMOTE in the study^85^ and DL model in^86^.

**Table 6:** Results of deep learning models^85^.

Model	Accuracy	Precision	Recall	F1 Score
ET-CNN	0.9524	0.97	0.97	0.97
CNN^86^	0.9398	0.95	0.95	0.95
MLP^86^	0.9120	0.94	0.94	0.94
RNN^86^	0.9100	0.89	0.91	0.90
LSTM^86^	0.8691	0.93	0.93	0.93
RF without SMOTE^85^	0.8889	0.89	0.89	0.89
ETC with SMOTE^85^	0.9262	0.93	0.93	0.93

In the study mentioned by Ishaq et al.^85^, Table 7 displays the accuracy results achieved by various models, including Decision Tree (DT), Logistic Regression (LR), Stochastic Gradient classifier (SGD), and others. The machine learning models utilized SMOTE oversampling techniques to give the best performance and to deal with class-imbalance problems. The comparison with the study^85^ shows the superiority of the proposed CNN model over all other best experiments done so far. Despite the utilization of SMOTE in their research^85^, the ET-CNN model outperformed these approaches.

**Table 7:** Performance comparison of ML and DL models.

Models	Accuracy
SGD^85^	0.5491
GNB^85^	0.7540
SVM^85^	0.7622
LR^85^	0.8442
DT^85^	0.8778
GBM^85^	0.8852
AdaBoost^85^	0.8852
RF^85^	0.9188
ETC^85^	0.9262
LSTM^86^	0.8691
RNN^86^	0.9100
MLP^86^	0.9120
CNN^86^	0.9398
ET-CNN	0.9524

### Performance comparison with transfer learning models

This study also conducted a performance comparison using transfer learning approaches. Two transfer-learning models, namely AlexNet and Visual Geometry Group (VGG 16), are employed for this analysis. The VGG-16 architecture comprises pooling, padding, connected, and convolution layers, while AlexNet is also a convolutional neural network (CNN) with a large number of attributes. Table 8 provides a performance evaluation comparing the VGG-16, CNN, and AlexNet models. The CNN model demonstrates superior performance, although the VGG-16 model also exhibits good precision, recall, and F1 scores.

**Table 8:** Proposed CNN performance comparison with transfer learning models.

Model	Accuracy	Precision	Recall	F1 Score
AlexNet	0.9170	0.89	0.89	0.89
VGG 16	0.9291	0.89	0.90	0.90
ET-CNN	0.9524	0.97	0.97	0.97

In Table 9, a comparison of the training times for the proposed CNN model and the transfer learning model is presented, providing insights into their computational complexity. Table 9 indicates the superiority of the proposed approach in terms of computational complexity. Tables 8 and 7 indicate the superiority in terms of experimental results performance.

**Table 9:** Training time complexities of the models.

Model	Time (min)
AlexNet	21
VGG-16	17
Proposed approach	10

### K-fold cross-validation results of the proposed model

The significance of the proposed model is checked by utilizing the cross-validation technique using the heart disease dataset. The proposed model based on ET-CNN successfully categorizes patient data, achieving 0.960, an average accuracy. Additionally, the recall, precision, and F1 scores for the model are reported as 0.973, 0.976, and 0.975, respectively. The result of k-fold cross-validation is shown in Table 10 using the proposed CNN model.

**Table 10:** Proposed model k-fold cross-validation.

Folds	Accuracy	Precision	Recall	F-Score
F1	0.953	0.971	0.978	0.974
F2	0.959	0.979	0.978	0.978
F3	0.961	0.975	0.975	0.975
F4	0.962	0.978	0.979	0.978
F5	0.959	0.979	0.982	0.980
Average	0.960	0.973	0.976	0.975

### Discussion

In the realm of managing large patient data, predominantly with critical heart failure requiring regular and uninterrupted observation, there is a growing tendency to have IoT-based intelligent patient monitoring systems. This research introduces a monitoring system based on AI and integrated with a machine-deep ET-CNN model for detecting heart failure instances. In order to ascertain the performance of our suggested ET-CNN prototypes against other transfer learning, deep learning, and ML models, numerous experiments were conducted during this study including AlexNet and VGG-16. The investigational conclusions reveal that the proposed ET-CNN model outperforms the other models, demonstrating the highest level of effectiveness, having a classification accuracy of 0.9524, the ET-CNN surpasses the performance of the alternative models employed in the study.

The proposed model demonstrates superior performance compared to all other deep learning and ML models. It achieves high recall, accuracy, precision, and F1 score results. The efficiency of the projected CNN model is further validated through 5-fold cross-validation, highlighting its efficacy. Additionally, a comprehensive comparison is conducted between the proposed approach and existing works, considering various characteristics such as the number of utilized features, the combination of AI methods, IoT integration, and monthly record-keeping of patients. The consequences of this comparison are illustrated in Table 11, which clearly indicates that the anticipated system outperforms existing approaches.

**Table 11:** The proposed system will be compared to existing systems using a “Yes” to indicate the presence of a feature and a “No” to indicate the absence of a specific feature.

Research works	Feature > 4	Patient history	AI learning technique	Cloud storage
^87^	No	No	Yes	Yes
^88^	No	No	Yes	Yes
^89^	No	No	Yes	Yes
^90^	No	No	Yes	Yes
^91^	No	No	Yes	Yes
^92^	No	No	No	Yes
^93^	No	No	No	Yes
^94^	No	No	No	Yes
Proposed model	Yes	Yes	Yes	Yes

### Comparison with published systems

Performance comparison of the proposed model with previously published paper on the same dataset. The comparison of accuracy between the proposed CNN model and the studies by^95–98^ is illustrated in Table 12^95^. Achieved an accuracy score of 0.85 for heart disease detection utilizing a modified logistic regression model. On the other hand, both both^96,98^employed the Naive Bayes (NB) model, obtaining accuracy scores of 0.74 and 0.86, respectively. In study^97^, a KNN model was used by the authors for the same objective and a higher accuracy of 0.92 was achieved. In contrast, the suggested model attained a 0.9524 score as far as the accuracy score is concerned, surpassing the performance of these studies.

**Table 12:** Results comparison with the previous systems.

Research studies	Models	Accuracy
Kumar Dwivedi^95^	Logistic regression	0.85
Parthiban et al.^96^	Naïve Bayes	0.74
Shah et al.^97^	K-NN	0.90
Vembandasamy et al.^98^	Naïve Bayes	0.86
Proposed Model	ET-CNN	0.9524

### Proposed system performance on the real-time dataset

To thoroughly assess the performance of the proposed approach, additional experiments were conducted using an instantaneously collected dataset, which allowed for the evaluation of the proposed model’s efficacy in a real-world setting, providing insights into its performance in practical scenarios.

#### Cleveland dataset

The public health dataset is utilized, containing four subsets namely Switzerland, Cleveland, Long Beach V, and Hungary. These datasets have 76 features but only 14 features out of these 76 features have been used in all the published research to date in this context. A dataset containing cardiac disease-related data from across the USA and 303 patients from Ohio state-based CCF (Cleveland Clinic Foundation). UCI_MLRepository is the cardiac disease-related publicly available dataset^99^. Every clinical case out of 303 includes a target attribute among a total of 76 features. The target attribute is represented by an integer ranging from 0 to 4, where 0 indicates a heart patient and values in the range of [1, 2, 3] represent healthy subjects. For the purpose of this study, binary classification was employed, having target values set to 0 for heart patients and 1 for healthy individuals. 125 instances out of 282 clinical sessions were found to be cardiac cases while the rest of the cases (55.67% or 157 out of 282) were healthy ones.

#### Results of the experiments

Table 13 provides the outcomes of real-time data based on deep learning models. Deep learning models are preferred over ML models due to their excellent performance in comparison. Among deep learning models, the projected ET-CNN model demonstrates significant performance with an accuracy value of 0.9718, outperforming MLP, RNN, and LSTM models. Following the CNN model, the RNN model achieves an accuracy score of 0.9449. In terms of F1 score and precision, the proposed CNN model outperforms the other models. It also outperformed the rest of the models in recall scores with the highest score of 0.98.

**Table 13:** Performance comparison of the deep learning models on real-time data.

Model	Accuracy	Precision	Recall	F1 Score
ET-CNN	0.9718	0.95	0.98	0.96
CNN^86^	0.9534	0.93	0.97	0.95
MLP^86^	0.9302	0.91	0.93	0.92
RNN^86^	0.9449	0.92	0.92	0.92
LSTM^86^	0.9329	0.91	0.92	0.91

### Results comparison with modern approaches

Our suggested healthcare monitoring method for cardiac patients is evaluated against the existing well-established and advanced methods in the domain. An ensemble method was used to develop a cardiovascular risk prediction system based on IoT, in a study referenced^100^. However, it is important to note that ensemble techniques can introduce higher complexity and computational resource requirements, which may not be suitable for health-related systems. Furthermore, if not properly implemented, these techniques can lead to overfitting issues. Table 14 compares the performance inter-alia proposed and referenced^100^ models. The results clearly demonstrate that the suggested approach is better than the mentioned study, showcasing its superior performance and effectiveness in health monitoring applications.

**Table 14:** Performance comparison of the proposed approach with existing research work^100^.

Voting classifiers	Precision (%)	Recall (%)	F1 Score (%)
KNN, ADA, XGB^100^	88.8	89.0	88.9
XBG, KNN, SVM^100^	87.8	87.8	87.8
MLPC, KNN, XGB^100^	91.0	91.0	91.0
KNN, ADA, MLPC, XGB^100^	87.1	87.5	87.3
MLPC, SVM, ADA, XGB^100^	88.2	88.6	88.4
SVM, XGB, MLPC, KNN, ADA^100^	87.2	87.3	87.2
CNN^86^	93.4	97.5	95.6
Proposed ET-CNN	95.1	98.24	96.3

### Limitations of current work

The major limitation of this research work is the collection of data from multiple sources and the sending of data to the cloud storage for further analysis. However IoT-based systems are capable for incorporation of digital wearable fitness gadgets that can be controlled via multiple devices by healthcare professionals. Such devices have limited processing powers and local data storage. Such devices are relatively less secure and hence a potential risk to the privacy and confidentiality of patients’ data. The integration of IoT-based wearable devices has the potential to provide continuous monitoring of patients, enabling early detection of potential health issues. This expanded system can enhance the overall healthcare monitoring process and contribute to the timely diagnosis and management of patient’s health conditions.

## Conclusions

Monitoring patients with acute heart holds significant importance in acting rapidly in case of emergencies. Incorporating IoT and artificial intelligence-based solutions has emerged as potential solutions in medical care. The utilization of IoT technology has enabled medical professionals to access patients’ records without requiring them to physically visit medical clinics. This study presents the development of a health monitoring system aimed at monitoring the health status of individuals with critical heart failure. The proposed healthcare system has increased the survival chances for chronically ill patients and offers convenient, cost-effective, and reliable monitoring specifically for cardiac patients. The system gathers data from patients and transmits it to the cloud for further analysis. Furthermore, this study introduces an extra tree classifier with an optimized convolutional neural network model for the precise identification of individuals with heart conditions. The performance of this model is evaluated in comparison to various deep learning, machine learning, and transfer learning approaches. The trial findings demonstrate that the suggested prototype outperforms all other utilized models, achieving an accuracy score of 0.9524. The effectiveness of the proposed model is further confirmed through 5-fold cross-validation. Furthermore, a comparison of its performance with existing studies that employed the same dataset. In both cases, the proposed model consistently exhibits superior results. Additionally, the proposed technique demonstrates higher accuracy and shorter training time compared to alternative models. The future work of this research work is to design an automated telemedicine system on which patients not only can continuously monitor healthcare but also can get proper medication based on health monitoring data.

## Data Availability

The dataset generated and analyzed during the current study are available in the Kaggle repository, (https://www.kaggle.com/datasets/mohamedamineferrag/edgeiiotset-cyber-security-dataset-of-iot-iiot). The dataset analyzed during the current study is available from the corresponding author upon reasonable request.

## Abbreviations

AI: Artificial Intelligence
WHO: World Health Organization
CNN: Convolutional Neural Network
LSTM: Long Short Term Memory
MLP: Multi-layer Perceptron
ANN: Artificial Neural Network
ETC: Extra Tree Classifier
SVM: Support Vector Machine
LR: Logistic Regression
RF: Random Forest
VC: Voting Classifier
GBM: Gradient Boosting Machine
SGD: Stochastic Gradient Descent
GNB: Gaussian Naive Bayes
YOLO: You Only Look Once
VGG: Visual Geometry Group
TN: True Negative
TP: True Positive
FN: False Negative
FP: False Positive
